# Wild-type transthyretin cardiac amyloidosis mimicking hypertrophic obstructive cardiomyopathy: A case report

**DOI:** 10.1007/s00508-025-02540-8

**Published:** 2025-05-13

**Authors:** Nikol Kubinova, Tomas Paleček, Marek Mika, Radek Jaksa, Ales Linhart

**Affiliations:** 1 2nd Department of Medicine—Department of Cardiovascular Medicine, First Faculty of Medicine, Charles University in Prague and General University Hospital in Prague, U Nemocnice 2, 128 08 Prague 2, Czech Republic; 2 Department of Internal Medicine, Hospital Strakonice, Strakonice, Czech Republic; 3 Institute of Pathology, First Faculty of Medicine, Charles University and General University Hospital in Prague, Prague, Czech Republic

**Keywords:** Amyloid cardiomyopathy, Echocardiography, Heart failure, Carpal tunnel syndrome, Polyneuropathy

## Abstract

**Background:**

Wild-type transthyretin cardiac amyloidosis (ATTRwt CA) is increasingly recognized as an important cause of heart failure and arrhythmias in older people. There are several clinical, echocardiographic, electrocardiographic (ECG) and laboratory features that increase the suspicion for ATTRwt CA. Presentation and phenotype can, however, be associated with atypical findings making it difficult to make a correct diagnosis.

**Case summary:**

A 65-year-old man was admitted for an acute coronary syndrome. Echocardiography revealed diffuse concentric left ventricular (LV) thickening. Because of a history of bilateral carpal tunnel syndrome and polyneuropathy, the patient underwent dedicated laboratory testing and diphosphonate scintigraphy the results of which were suggestive of transthyretin cardiac amyloidosis. Also, a dynamic LV outflow tract obstruction due to the systolic anterior motion of the anterior mitral valve was noted on echocardiography during the initial investigations. Genetic testing for hypertrophic cardiomyopathy was negative. Seeking a conclusive diagnosis, endomyocardial biopsy was performed. This confirmed the diagnosis of ATTRwt CA.

**Discussion:**

The presence of dynamic LV outflow tract obstruction is typically seen in patients with sarcomeric hypertrophic cardiomyopathy. It can be rarely seen also in individuals with cardiac amyloidosis, including ATTR-wt CA. The presence of so-called red flags in patients’ history, physical examination, laboratory test, ECG and imaging should raise suspicion for other etiologies of LV wall thickening than hypertrophic cardiomyopathy. Although noninvasive diagnosis of ATTRwt CA is possible in most patients, endomyocardial biopsy remains necessary in cases with diagnostic ambiguity.

## Introduction

Cardiac amyloidosis is a progressive disease characterized by the extracellular deposition of insoluble fibrillar proteins within the heart. There are predominantly two major forms that affect the heart: light chain (AL) and transthyretin-related (ATTR) amyloidosis, the latter can be secondary to wild-type ATTR amyloidosis (ATTRwt) or its hereditary form (ATTRv) [1].

Amyloid heart disease typically manifests as left ventricular wall thickening associated with progressive diastolic dysfunction. Cardiac amyloidosis must be distinguished from other heart diseases associated with left ventricular (LV) wall thickening including hypertrophic cardiomyopathy (HCM) [1]. Unlike HCM, where the left ventricular outflow tract (LVOT) is obstructed in 30% of patients and provoked in 70% of cases, LVOT obstruction (LVOTO) in amyloid cardiomyopathy is a rare finding [2]. We present an interesting case of a patient with ATTRwt and dynamic LVOTO.

## Case presentation

**Fig. 1:**
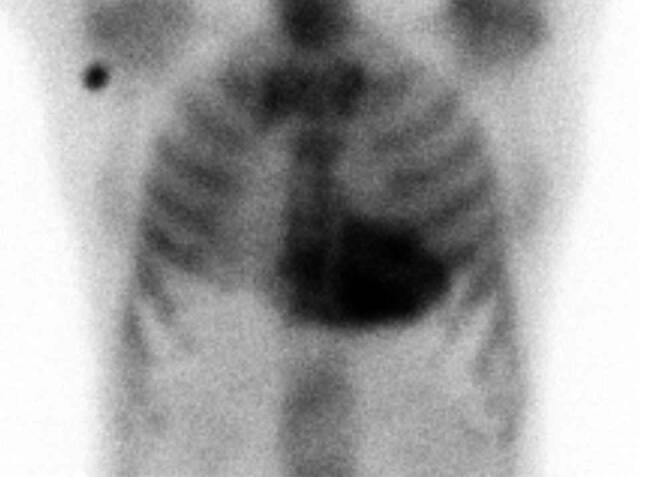
^99m^Tc‑3,3‑diphosphono‑1,2‑propanodicarboxylic acid (99mTc-DPD) bone scintigraphy: anterior planar image demonstrating intense myocardial tracer uptake (Perugini grade 3)

A 65-year-old man was initially admitted to a regional hospital in March 2023 for typical anginal chest pain lasting 3 h. A non-ST elevation acute coronary syndrome was diagnosed and the patient underwent successful percutaneous coronary intervention of a diagonal branch with a drug-eluting stent implantation. His past medical history included arterial hypertension and hyperlipidemia. Of note, there was also a 3-year history of unoperated bilateral carpal tunnel syndrome and 1‑year history of peripheral polyneuropathy. There was no history of spontaneous biceps tendon rupture, lumbar or cervical spinal stenosis. During hospitalization, an echocardiography revealed significant concentric left ventricular (LV) hypertrophy with no regional wall motion abnormalities, preserved LV ejection fraction and grade II diastolic dysfunction. Based on these findings together with the history of abovementioned extracardiac red flags, cardiac amyloidosis was suspected. The use of ^99m^Tc‑3,3‑diphosphono‑1,2‑propanodicarboxylic acid (99mTc-DPD) bone scintigraphy demonstrated high-grade cardiac uptake (Perugini grade 3) (Fig. 1). Plasma cell dyscrasia was excluded by serum free light chain assay and by serum and urine protein electrophoresis with immunofixation. The combination of clearly positive scintigraphy together with negative assessments for monoclonal protein enabled the noninvasive diagnosis of transthyretin cardiac amyloidosis (ATTR-CA) and the patient was referred to our specialized amyloidosis center for further evaluation and management.

**Fig. 2:**
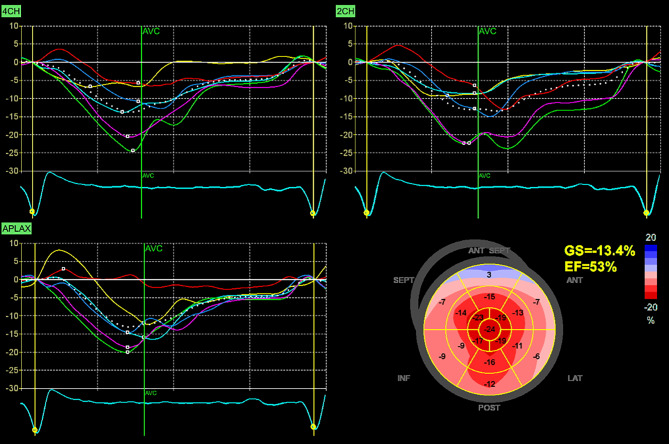
Echocardiography: Two-dimensional strain analysis showing significant reduction of global longitudinal left ventricular strain together with the relative apical sparing pattern

**Fig. 3:**
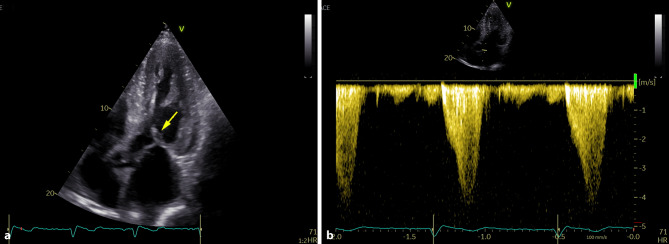
Echocardiography: **a** apical five-chamber view demonstrating systolic anterior motion of the anterior mitral valve leaflet (*yellow arrow*) causing dynamic left ventricular outflow tract obstruction; **b** continuous wave Doppler tracing showing typical “dagger-shaped” velocity signal of dynamic left ventricular outflow tract obstruction with resting gradient of 70 mm Hg

**Fig. 4:**
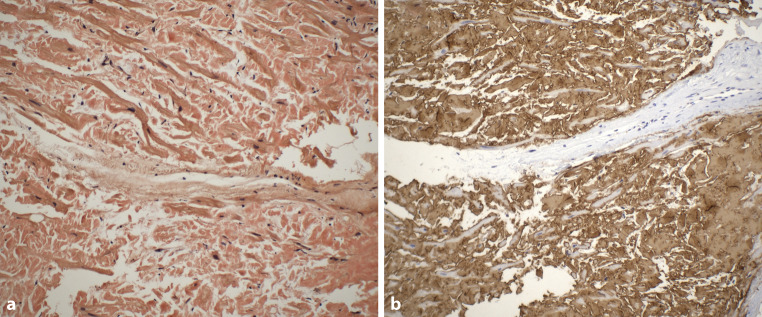
Endomyocardial biopsy histopathology. **a** Congo red stain showing abundant extracellular amyloid deposition (200×); **b** immunohistochemical analysis (performed in a BenchMark ULTRA automated stainer [Roche Ventana Diagnostics, Oro Valley, USA] demonstrating the presence of TTR amyloid (400×). Immunohistochemical staining for kappa and lambda light chains as well as for serum amyloid A was negative

During the first visit at our center in June 2023, the patient reported chronic shortness of breath consistent with functional class NYHA II, without any other symptoms of heart disease. His medications consisted of acetylsalicylic acid 100 mg once daily, clopidogrel 75 mg once daily, bisoprolol 5 mg daily, perindopril 5 mg daily and atorvastatin 20 mg daily. Blood pressure was 125/70 mm Hg, heart rate was 73 beats/min, respiratory rate was 16 respirations/min and oxygen saturation was 96% in room air. A holosystolic murmur at the left upper sternal border represented the main finding during the physical examination. Lung auscultation revealed clear breath sounds bilaterally. No signs of volume overload were present. An electrocardiogram (ECG) demonstrated sinus rhythm with first degree atrioventricular block and left anterior fascicular block. Laboratory tests did not reveal any significant abnormalities except for an elevation in NT-pro-BNP level (1137 ng/l) together with mild elevation of high-sensitive TnI level (51 ng/l). Echocardiographic examination confirmed the presence of pronounced concentric, diffuse LV wall thickening (septal thickness 17 mm, posterior wall thickness 15 mm, LV end-diastolic diameter 44 mm, relative wall thickness 0.68) with the absence of regional wall motion abnormalities and preserved LV ejection fraction of 71%, grade II diastolic dysfunction and left atrial dilatation. Global longitudinal strain was significantly reduced (−13.4%), with the relative apical sparing pattern (Fig. 2). Systolic anterior motion of the mitral valve leaflets was present causing significant dynamic LV outflow tract obstruction (LVOTO) with peak gradient of 70 mm Hg at rest (Fig. 3) and 140 mm Hg with a Valsalva manoeuvre. Due to this unexpected finding we decided to perform an endomyocardial biopsy for a definitive diagnosis of ATTR-CA. The biopsy revealed abundant extracellular amyloid deposits stained with Congo red. Subsequent immunohistochemical analysis demonstrated the presence of ATTR (Fig. 4). Additionally, myocyte hypertrophy and myocardial disarray were not present. The final diagnosis of cardiac ATTRwt1 was established after negative genetic testing excluding ATTRv. No pathogenic or likely pathogenic variant associated with sarcomeric HCM or Fabryʼs disease were found. Specific disease-modifying therapy with the transthyretin stabilizer tafamidis (61 mg per day) was initiated. Bisoprolol was increased up to 10 mg daily which led to resolution of the LVOTO and an improvement in functional capacity to NYHA class I.

## Discussion

Compared to sarcomeric HCM, LVOTO represents a rare finding in amyloid heart disease. Dinwoodey et al. published a series of 97 consecutive patients with AL cardiac amyloidosis diagnosed in a 2-year period with the prevalence of LVOTO being 4% [3]. Only two case reports of ATTR-CA with LVOTO have been published by the end of 2023 [4, 5]. Notably, in the two largest studies describing echocardiographic findings in patients with ATTRwt CA, LVOTO was not reported in any case [6, 7]. More recently, Longinow et al. published the largest study so far focused on the prevalence of LVOTO in amyloid heart disease [8]. In 1299 consecutive patients with AL or ATTR-CA, LVOTO was observed in 36 individuals (2.7%), 58% of which had ATTR-CA. Of those with LVOTO, 58% underwent septal myectomy. Patients with ATTR-CA were older and more likely to undergo myectomy than those with AL amyloid heart disease.

Interestingly, Lamke et al. examined histopathological features of myocardial specimens of 204 consecutive patients with sarcomeric HCM undergoing septal myectomy [9]. These authors found amyloid deposits in 3 male individuals older than 68 years of age; in all cases immunohistochemical staining for ATTR was positive.

Our case nicely illustrates that even in the presence of echocardiographic finding of LVOTO, a thorough and comprehensive approach to the LV hypertrophy phenotype is necessary to elucidate its possible specific etiology. The basis of this approach represents the recognition of diagnostic red flags in the medical history, physical examination, routine laboratory tests, electrocardiogram, and imaging modalities [10, 11]. The recognition of the exact etiology of LV thickening is crucial to deliver the appropriate treatment including disease-modifying therapy which can improve clinical outcomes.

## Conclusion

Although LVOTO represents a common finding in patients with sarcomeric HCM, it can also be identified in patients with other etiologies of LV wall thickening including ATTRwt CA. A thorough approach to assessing LV hypertrophy phenotype with a focus on the presence on diagnostic red flags is essential for revealing the specific etiology of cardiomyopathy even in the presence of atypical findings like LVOTO.
